# The Interplay of Bottom-Up Arousal and Attentional Capture during Auditory Scene Analysis: Evidence from Ocular Dynamics

**DOI:** 10.1523/JNEUROSCI.0811-25.2025

**Published:** 2026-01-05

**Authors:** Mert Huviyetli, Maria Chait

**Affiliations:** ^1^Ear Institute, University College London, London WC1X 8EE, United Kingdom; ^2^Faculty of Health Sciences, Izmir Bakircay University, Izmir 35665, Türkiye

**Keywords:** auditory scene analysis, change detection, eye-tracking, microsaccades, predictive coding, pupil dilation

## Abstract

The auditory system plays a crucial role as the brain's early warning system. Previous work has shown that the brain automatically monitors unfolding auditory scenes and rapidly detects new events. Here, we focus on understanding how automatic change detection interfaces with the networks that regulate arousal and attention, measuring pupil dilation (PD) as an indicator of listener arousal and microsaccades (MS) as an index of attentional sampling. Naive participants (*N* = 36, both sexes) were exposed to artificial “scenes” comprising multiple concurrent streams of pure tones while their ocular activity was monitored. The scenes were categorized as REG or RND, featuring isochronous (regular) or random temporal structures in the tone streams. Previous work showed that listeners are sensitive to predictable scene structure and use this information to facilitate change processing. Scene changes were introduced by either adding or removing a single tone stream. Results revealed distinct patterns in the recruitment of arousal and attention during auditory scene analysis. Sustained PD was reduced in REG scenes compared with RND, indicating reduced arousal in predictable contexts. However, no differences in sustained MS activity were observed between scene types, suggesting no differences in attentional engagement. Scene changes, though task-irrelevant, elicited PD as well as MS suppression, consistent with automatic attentional capture and increased arousal. Notably, only MS responses were modulated by scene regularity. This suggests that changes within predictable environments more effectively recruit attentional resources. Together, these findings offer novel insights into how automatic auditory scene analysis interacts with neural systems governing arousal and attention.

## Significance Statement

Even without active listening, our brains automatically respond to changes in complex sound environments, like noticing a new sound on a busy street. These responses involve shifts in arousal and attention, helping us decide how to react, often without conscious awareness. Understanding this process is key to studying how we perceive sound scenes and how it may be disrupted in individuals with attention or arousal difficulties. In this study, participants passively listened to artificial soundscapes while we tracked eye activity: pupil dilation (a sign of arousal) and microsaccades (tiny eye movements linked to attention). We found that sudden scene changes triggered both responses, but they were differently influenced by scene predictability, suggesting they reflect separate aspects of automatic auditory processing.

## Introduction

The ability to rapidly respond to new events in our environment is crucial for survival. It is hypothesized that the auditory system functions as the brain's “early warning system,” continuously monitoring the unfolding acoustic environment to quickly direct attention to new events (Murphy et al., 2013; Winkler and Denham, 2024). Indeed, listeners are highly sensitive to abrupt changes—such as the appearance or disappearance of a sound source—even within complex, crowded auditory scenes (Eramudugolla et al., 2008; Pavani and Turatto, 2008; Cervantes Constantino et al., 2012; Puschmann et al., 2013; Petsas et al., 2016). Notably, brain responses, recorded from naive distracted listeners, indicate that such changes are often detected even in the absence of directed attention (Sohoglu and Chait, 2016a,b; Poole et al., 2025), supporting the notion that auditory change detection is, at least in part, an automatic process.

A key factor enhancing this ability is the brain's sensitivity to predictable structure in the environment. Change detection performance improves significantly when sound streams follow a predictable pattern compared to when they fluctuate randomly (Sohoglu and Chait, 2016a; Aman et al., 2021; de Kerangal et al., 2021; Zuk et al., 2025). These findings align with a broader body of research demonstrating that the brain is finely attuned to statistical regularities in sensory input and exploits this information to enable efficient interaction with its surroundings (Winkler et al., 2009; Bendixen, 2014; Barascud et al., 2016; de Lange et al., 2018).

The general understanding emerging from these investigations suggests that the auditory system continuously monitors for changes in unfolding scenes, relaying this information to attention and arousal networks to initiate an appropriate response (e.g., fight or flight). Attention and arousal are related but dissociable systems. Arousal denotes the global physiological and psychological state of alertness or activation (Samuels and Szabadi, 2008). In contrast, attention refers to mechanisms that prioritize sensory processing, ranging from voluntary, goal-directed selection to involuntary, “bottom-up” stimulus-driven capture (Corbetta and Shulman, 2002). In the present study, attention is considered in terms of automatic attentional capture, whereby unexpected or salient changes in the auditory scene transiently recruit processing resources without task relevance. Bottom-up driven attention and arousal interact closely: arousal shapes the efficacy of attentional capture, while attention can modulate arousal (Petersen and Posner, 2012; Maness et al., 2022). At the circuit level, the locus ceruleus (LC), which supplies the brain with the neurotransmitter norepinephrine (NE) that regulates vigilance and arousal, interacts with networks that control attention, with NE playing a central role in tuning the gain of circuits that implement attentional processes (Torres et al., 2025).

We examined how automatic change detection interfaces with these neural systems by recording eye and pupil dynamics as participants listened to “artificial acoustic scenes” (see Materials and Methods). Specifically, we analyzed pupil dilation (PD) and pupil dilation rate (PDR), as indices of arousal (Joshi and Gold, 2020), and microsaccades (MS), as markers of attentional sampling (Pastukhov and Braun, 2010; Schneider et al., 2020; Zhao et al., 2024; Liu and Chait, 2025). Pupil size is a widely used proxy for LC activity: baseline PD reflects tonic LC activity linked to general alertness, whereas transient dilations index phasic activations signaling rapid arousal to novel stimuli (Aston-Jones and Cohen, 2005; Joshi et al., 2016; Wang and Munoz, 2021). MS are small, involuntary fixational eye movements thought to support automatic environmental exploration (Martinez-Conde et al., 2006; Otero-Millan et al., 2008). Novel stimuli transiently suppress MS (microsaccadic inhibition, MSI; Engbert and Kliegl, 2003; Rolfs et al., 2008), a process modulated by stimulus salience and attention (White and Rolfs, 2016; Roberts et al., 2019; Zhao et al., 2019b). MSI is thus considered to reflect a fast, adaptive attentional re-orienting mechanism that interrupts ongoing processing to prioritize novel inputs and guide appropriate behavioral responses.

Consistent with an interplay between arousal and attention, the circuits controlling MS and PD are interconnected, including reciprocal connections between the LC, the frontal eye fields (FEF) and superior colliculus (SC), which control MS (Hafed et al., 2009; Joshi and Gold, 2020; Wang et al., 2020). Yet growing evidence suggests that PD and MS capture dissociable processes (Contadini-Wright et al., 2023; Zhao et al., 2024; Liu and Chait, 2025), offering complementary windows into how these networks are engaged during specific tasks. We tested how novel auditory events recruit these pupil- and MS-linked systems, to shed light on the mechanisms that support auditory scene analysis and situational awareness.

## Materials and Methods

### Ethics

The research was approved by the Research Ethics Committee of University College London. Participants provided written informed consent and were paid for their participation.

### Participants

Thirty-six paid participants (26 female; mean age: 23.5, range: 18–34, SD = 4.58) were recruited. All reported normal hearing with no history of otological or neurological disorders and normal or corrected-to-normal vision, with SPH prescriptions no higher than 3.5. One participant was excluded from the pupil diameter and microsaccade analysis due to exceedingly long reaction times on the decoy task (see below); three participants were excluded from pupil diameter and microsaccade analysis because of difficulty tracking the eye or excessive blinking or tiredness; three participants were excluded from microsaccade data analysis due to nonavailability of binocular recording (see below for information on detecting microsaccadic activity).

### Stimuli

**Figure 1.**
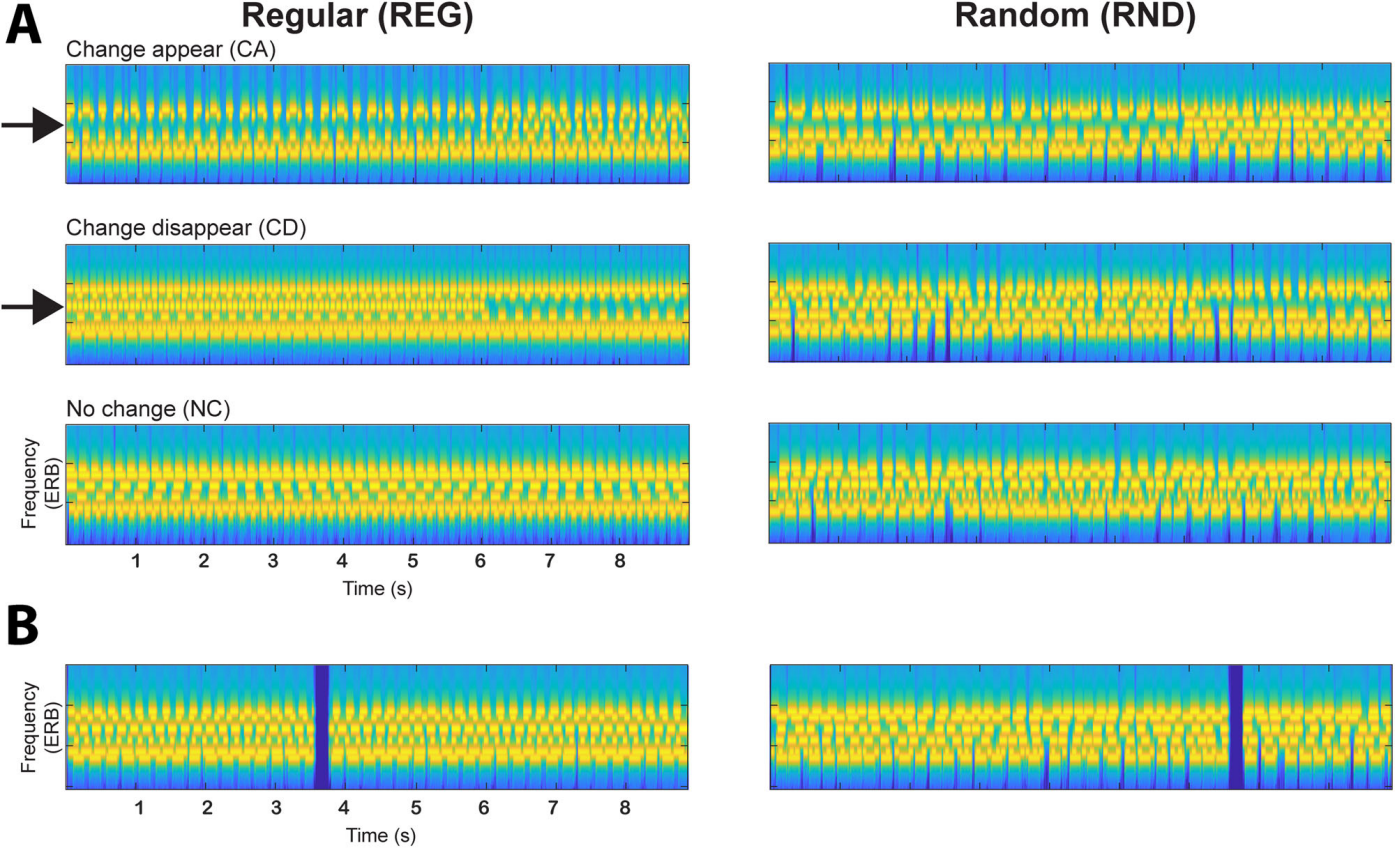
Change detection paradigm. ***A***, Example of the three variations (“change appear,” “change disappear,” and “no change”) of a scene with six streams. Regular (REG) scenes are on the left, and random (RND) scenes are on the right. The changing component is indicated with an arrow. ***B***, Examples of gap-containing scenes.

Stimuli (Fig. 1) were largely identical to those used in Aman et al. (2021), except they were made longer (9 s long instead of 4 s long) to accommodate the expected slower pupil responses. “Scenes” were populated by six streams of pure tones, representing six concurrent sound sources. Each stream had a unique carrier frequency [chosen from a pool of nine fixed values between 500 and 3,225 Hz, spaced 2 cams on the ERB scale; “Equivalent Rectangular Bandwidth”; Moore and Glasberg (1983) and a unique temporal structure]. These “scenes” were thus perceived as composite “soundscapes”, in which individual streams are perceptually separable. As such, they serve as effective models for busy natural acoustic environments (Cervantes Constantino et al., 2012). In “regular” (REG) scenes, the streams had a regular temporal structure: for each stream, tone pip duration and intertone interval duration were each chosen randomly from between 10 and 150 ms and then fixed for the duration of the scene. This yielded “sources” with a variety of rates (from 3 to 50 Hz) spanning the range which characterizes speech (Rosen, 1992) and many natural sounds. In “random” (RND) scenes, the streams had a random temporal structure: for each stream, tone pip duration was chosen randomly from the values above and fixed for the duration of the scene, but the intertone interval varied (also chosen from the range above) to yield an irregular temporal pattern. All streams (in REG or RND scenes) were phase randomized [such that they started with the tone or the silent intertone interval (ITI)]. Each tone was ramped on and off with a 3 ms raised cosine ramp.

Scenes in which each source is active throughout are referred to as “no change” (NC) scenes. In “change appear” (CA) scenes, a single stream is added partway through the scene.

In “change disappear” (CD) scenes, a single stream is deleted partway (Fig. 1). The change (appearing or disappearing) component was chosen randomly for each scene. The timing of the change in CA scenes, defined as the time at which the first tone pip of the appearing stream was presented, was 6 s after scene onset. For CD in REG scenes, the time of change was set to the offset of the last tone augmented by the intertone interval, i.e., at the expected onset of the next tone, which is the earliest time at which the disappearance is theoretically detectable. Therefore, change time varied somewhat from scene to scene but was always ∼6 s post-onset. For CD in RND scenes, change time is ill-defined (because the temporal pattern is random). Hence, following the approach adopted in Aman et al. (2021) and de Kerangal et al. (2021), the change time in those scenes was set to the offset of the last tone augmented by the mean intertone interval (80 ms).

The stimulus set also included “decoy” (task-relevant) scenes (20% of the stimuli) that contained a 200 ms silent gap that the participants were instructed to detect and respond to as soon as possible. The gap was inserted randomly anywhere between 1 s post onset to 1 s pre offset. The task served the purpose of keeping the participants alert and broadly engaged with the auditory stimuli but was calibrated to be easy so that it minimally draws on attention/computational resources.

Overall, the main experiment consisted of eight ∼7-min-long blocks, each containing 30 trials: four trials of each of the main conditions REG-CA, REG-CD, REG-NC, RND-CA, RND-CD, and RND-NC and one trial of each condition containing a gap. The stimuli were presented in random order. In total, 32 trials of each of the main conditions were available for analysis. The decoy (gap-containing) trials (48 overall) or any other trials that contained a button press (“false alarm”) were not included in the analysis of the ocular data.

The experimental session lasted ∼2 h and was composed of two stages:

1. Baseline ocular measures: Prior to the main experimental session, we performed a series of brief baseline measures of ocular reactivity. These included measuring responses to a slow, gradual change in screen brightness, to a sudden flashing white screen, to a sudden flashing black screen, and to a sudden presentation of a brief auditory stimulus (harmonic tone). These measurements are used to confirm normal ocular reactivity.
2. Main experiment: In the main experiment, ocular data were recorded while participants listened to the artificial scene stimuli and performed the decoy gap detection task. A short practice was provided beforehand to ensure participants understood the task. Participants were naive to the experimental conditions (scene changes and scene predictability) and were instructed to monitor for and quickly respond to the silent gaps. On trials on which a response was made (a correctly detected gap or a false alarm), feedback was provided. A summary of performance was also presented at the conclusion of each block. Approximately 3 min breaks were provided between blocks. Eight blocks were completed.

All experimental tasks were implemented in MATLAB and presented via Psychophysics Toolbox Version 3 (PTB-3).

#### Procedure

Participants sat with their head fixed on a chinrest in front of a monitor (24 inch BENQ XL2420T with a resolution of 1,920 × 1,080 pixels and a refresh rate of 60 Hz) in a dimly lit and acoustically shielded room (IAC triple walled sound-attenuating booth). They were instructed to continuously fixate on a black cross presented at the center of the screen against a gray background with a measured luminance of ∼83 cd/m^2^ (measured from the chinrest position using a Konica Minolta LS-150 luminance meter). An infrared eye-tracking camera (EyeLink 1000 Desktop Mount, SR Research) placed below the monitor at a horizontal distance of 62 cm from the participant was used to record eye data. Auditory stimuli were delivered diotically through a Roland Tri-Capture 24 bit 96 kHz soundcard connected to Sennheiser HD558 headphones. The loudness of the auditory stimuli was adjusted to a comfortable listening level for each participant (∼63 ± 3 dB SPL). The standard five-point calibration procedure for the EyeLink system was conducted prior to each experimental block, and participants were instructed to avoid any head movement after calibration. During the experiment, the eye-tracker continuously tracked gaze position and recorded pupil diameter, focusing binocularly at a sampling rate of 1,000 Hz. Participants were instructed to blink naturally during the experiment and encouraged to rest their eyes briefly during intertrial intervals. Prior to each trial, the eye tracker automatically checked that the participants’ eyes were open and fixated appropriately; trials would not start unless this was confirmed.

### Analysis of behavioral data

#### Gap detection task

Key presses that occurred <2 s following a target gap were designated as hits. We also tracked the number of false alarms (responses when no gap was present) and reaction times (recorded from each hit). Related-samples Wilcoxon signed rank test was conducted to test the main effect of regularity (REG and RND) on gap detection. Overall, participants made few false alarms; therefore, only hit rate and reaction time were analyzed.

### Pupillometry preprocessing and analysis

Trials containing a gap or where a participant made a false alarm were excluded from the analysis. Where possible, data from the left eye were analyzed. Intervals where the participant gazed away from fixation (outside of a radius of 100 pixels around the center of the fixation cross) or where full or partial eye closure was detected (e.g., during blinks) were automatically treated as missing data (including 250 ms before/after each eye closure event to account for blink-edge artifacts). All missing data segments were recovered using shape-preserving piecewise cubic interpolation. Data were then smoothed with a 150 ms Hanning window.

To focus on sequence evoked responses, the pupil data (NC trials) were epoched from 1 s before stimulus onset to 1 s post offset (−1: 10 s). To focus on change-evoked effects, the pupil data (CA and CD trials, with NC as control) were epoched from 0.5 s before change time to 2.5 s after change time (−0.5: 2.5 s). Epochs with >50% missing data or those determined to be particularly noisy where 10% or more of the data were identified as outlying (>3 SD from the condition mean) were discarded from the analysis. On average, <6 trials were discarded per subject for all conditions. Data were normalized to allow for comparison across trials and subjects. To do this, within each subject, the mean and standard deviation across all baseline samples (1 s for the sequence evoked analysis and 0.5 s for change-locked response analysis) in each block were calculated for each condition and used to *z*-score normalize the relevant epoched data. Thereafter, pupil diameter was time domain averaged across all epochs to produce a single time series per condition per subject.

### Pupil dilation rate analysis

To derive the pupil dilation rate (PDR) time series, pupil events were extracted from the continuous, smoothed data (150 ms Hanning window). Based on Joshi et al. (2016) and Zhao et al. (2019a, 2024), the events were defined as local minima that are followed by continuous dilation of the pupil for at least 100 ms. In each condition, for each participant, the event time series were summed and normalized by the number of trials and the sampling rate. Then, a causal smoothing kernel $\omega (\tau )=\alpha^{2}\times \tau \times e^{-\alpha \tau }$ was applied with a decay parameter of $\alpha =\frac{1}{50}\mathrm{ms}$ (Dayan and Abbott, 2005; Rolfs et al., 2008; Widmann et al., 2014) paralleling a similar technique for computing neural firing rates from neuronal spike trains (Dayan and Abbott, 2005; see also Rolfs et al., 2008; Joshi et al., 2016). The mean across trials was computed and baseline corrected. To account for the time delay caused by the smoothing kernel, the time axis was shifted by the latency of the peak of the kernel window.

### Microsaccade preprocessing and analysis

Intervals, where full or partial eye closure was detected (e.g., during blinks), were automatically treated as missing data and not interpolated. Microsaccade (MS) detection was based on an approach proposed by Engbert and Kliegl (2003). MS were extracted from the continuous eye-movement data based on the following criteria: (1) a velocity threshold of *λ* = 6 times the median-based standard deviation within each block; (2) above-threshold velocity lasting between 5 and 100 ms; (3) the events are detected in both eyes with onset disparity <10 ms; and (4) the interval between successive microsaccades is longer than 50 ms. Extracted microsaccade events were represented as unit pulses (Dirac delta). For sequence evoked responses, the data were epoched from 0.5 s before stimulus onset to 1 s post offset (−0.5: 10 s), and for change-evoked responses, the data were epoched from −0.15 s before change time to 2.5 s after change time (−0.15: 2.5 s). Epochs with >50% missing data were discarded from the analysis. The microsaccade rate was then computed in the same way as described for the pupil dilation rate, above.

We used shorter baseline windows, see above, in the MS analysis compared with the PD analysis, as the noisier nature of MS data required a briefer, more stable baseline period to ensure comparable amplitude alignment at the transition point.

### Statistical analysis

To identify time intervals showing significant PD/MS differences between conditions, we employed a nonparametric, bootstrap-based statistical analysis (Efron and Tibshirani, 1994). For each participant, we computed the difference time series between conditions and subjected these to bootstrap resampling (1,000 iterations with replacement). At each time point, differences were considered significant if >95% of the bootstrap iterations fell consistently above or below zero. This analysis was conducted across the full epoch.

The robustness of these effects was assessed with a bootstrap-based control analysis. For sequence evoked (sustained) PD/MS effects, the NC condition served as the control; for phasic PD comparisons with NC (CA–NC, CD–NC), the NC condition was used as a control; and for the CA–CD comparison, the CD condition was used as a control. In each bootstrap iteration, control condition trials for each participant were randomly split into two surrogate sets and compared, yielding a distribution of the longest spurious significant interval. This distribution defined the noise floor: any observed effect exceeding it was deemed significant (Supplementary Materials). As shown in Figures S1 and S2, significant effects remained detectable even with half the trials, underscoring their robustness. For MSI and PDR effects, we did not apply false discovery rate (FDR) correction. Instead, significant effects outside the predefined region of interest (100–250 ms, the typical MSI latency window) were treated as exploratory.

## Results

### Gap detection performance

**Figure 2.**
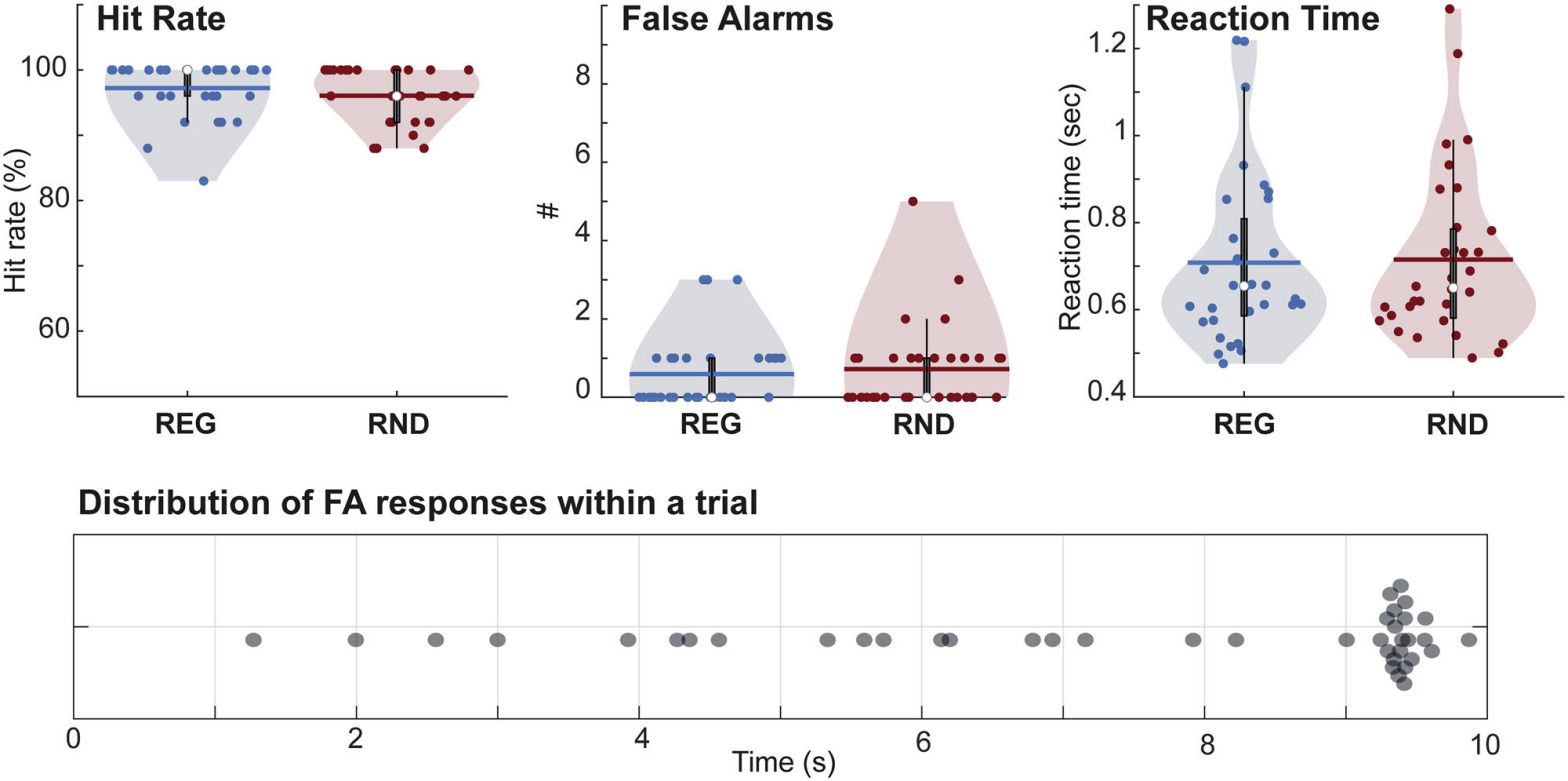
Decoy (gap detection) task performance. Dots represent individual data. Error bars show SEM. No difference was observed in terms of RTs and hit rate between REG and RND conditions. The bottom panel depicts the within-trial distribution of false alarms (collapsed across participants); each false alarm event is indicated by a dot. Trial onset is at 0 s and offset is at 9 s. The number of false alarm events was very low overall, with false alarms usually occurring following trial offset, consistent with a confusion between sound offset and the target silent gap.

As expected, the decoy gap detection task was easy (Fig. 2). Hit rates were high and close to ceiling in most participants. False alarm rates were very low across participants (<5 false alarms per subject across the full session). Pairwise Wilcoxon signed-rank tests were conducted to compare Hit rate and reaction time data across conditions. There was no difference between REG and RND conditions in terms of hit rate (*Z* = −1.458, *p* = 0.145) or reaction time (*Z* = −1.228, *p* = 0.219).

Figure 2 (bottom) shows the time course of false alarm responses (collapsed across participants) within a trial. False alarms were uniformly distributed across the epoch but peaked following trial offset (at 9 s), consistent with the similarity between sound cessation and the task-defined target (a silent gap). The pattern indicates no measurable confusion with scene changes at 6 s post-onset. Whereas gap detection relies on brief reductions in overall scene loudness, scene changes involve negligible loudness variation; instead, previous work has demonstrated that they are detected through second-order transients reflecting changes in activation within specific frequency bands (see more detail in Cervantes Constantino et al., 2012).

### Pupil dilation and microsaccade data reveal re-orienting responses to task-irrelevant scene changes

**Figure 3.**
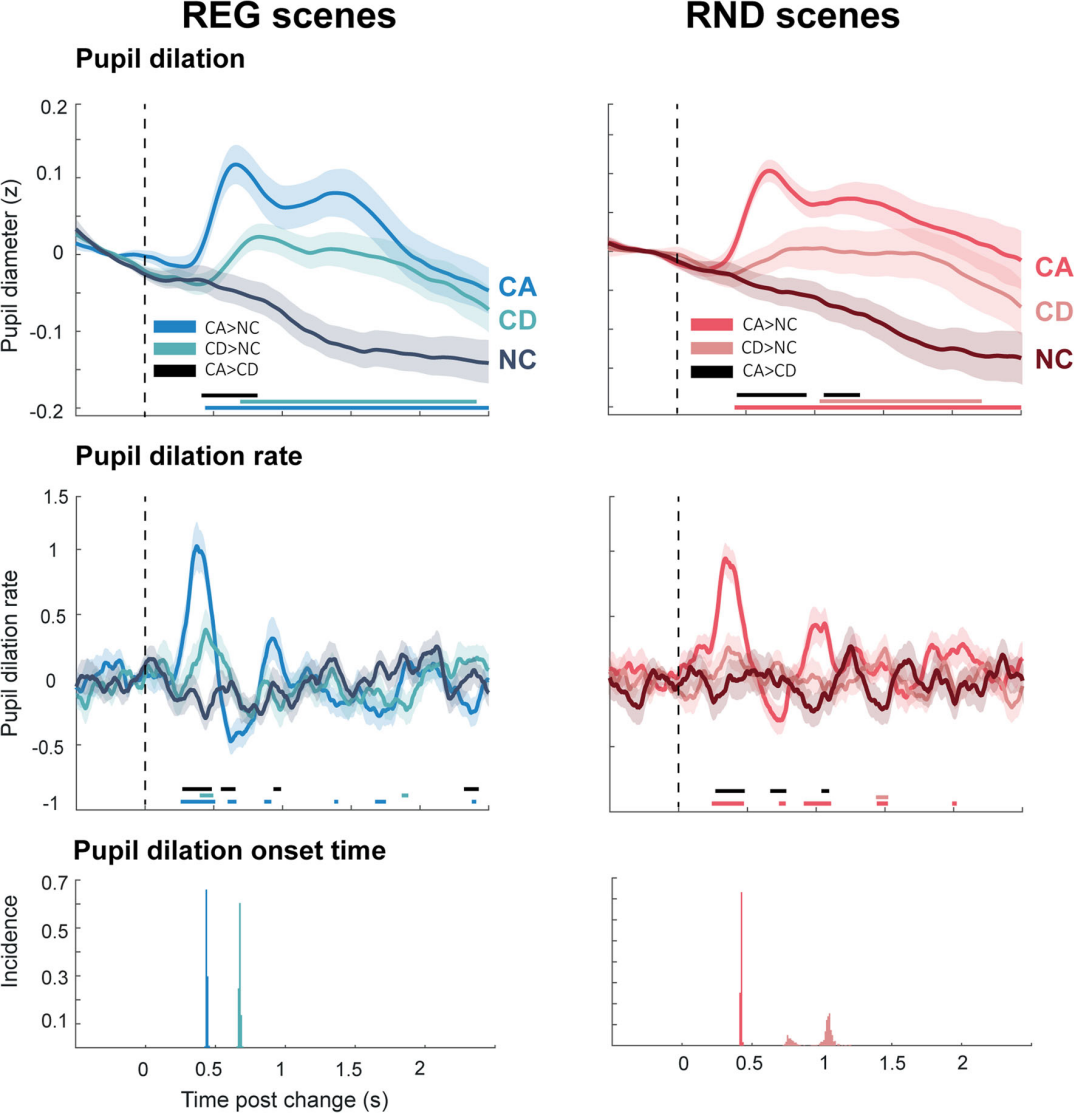
Change-evoked pupil dilation (PD; top) and pupil dilation rate (PDR; middle) across conditions [change appearance (CA), change disappearance (CD), no change (NC)]. Baseline corrected (−0.5 to 0 s pre-onset for PD and −0.15 to 0 s pre-onset for PDR). Shading indicates SEM. The horizontal bars indicate significant differences between conditions (*p* < 0.05; see legend within the figure). The bottom panel shows resampling-derived distributions of pupil dilation divergence latency between change CA/CD and NC. See Figure S1 for further control analyses confirming the robustness of the PD data. Effect sizes (Wilcoxon *r*) were calculated from the mean score at significant time points using nonparametric Wilcoxon signed-rank tests. For PD responses, effect sizes ranged between *r* = 0.68–0.40 across REG and RND conditions (REG: 0.63, 0.54, 0.53; RND: 0.68, 0.40, 0.57), respectively, reflecting CA > NC, CD > NC, and CA > CD. For PDR responses, effect sizes ranged between *r* = 0.74–0.42 (REG: 0.74, 0.52, 0.42; RND: 0.70, 0.51), respectively, showing CA > NC, CD > NC, and CA > CD for REG and CA > NC and CA > CD for RND. Note that for the PDR analysis, we focused on the early significant time points up to 500 ms after the change.

Figure 3 shows PD responses to scene changes against the no-change control (NC). Responses to both CA and CD (in both REG and RND scenes) show a pronounced increase in pupil diameter, consistent with a phasic response. The dynamics of these responses mirror dynamics observed in MEG data (Sohoglu and Chait, 2016b) a sharp early double-peaked response evoked by CA and a later, single-peaked response evoked by CD.

To assess potential latency differences between conditions, we conducted a resampling-based analysis to estimate the distribution of divergence latencies across comparisons (CA vs NC, CD vs NC) within each regularity condition (REG and RND). In each of 500 iterations, *N* = 32 participants were sampled with replacement, and the latency of the first significant difference between each condition pair was calculated using the same bootstrap procedure as in the main analysis (see Materials and Methods). The resulting distributions (Fig. 3, bottom) revealed mean divergence latencies of ∼450 ms for CA (in both REG and RND conditions). For CD, mean latencies were ∼670 ms in REG and ∼1,000 ms in RND. These findings confirm that PD responses emerge earlier in CA than in CD, and, within CD, earlier in REG than in RND scenes.

We also specifically focused on pupil-dilation rate (see Materials and Methods), as a potentially more sensitive measure of phasic pupil activity, which is associated with corresponding phasic activity in the Locus Coeruleus (Joshi et al., 2016; see also Jagiello et al., 2019; Zhao et al., 2024). These results revealed a consistent pattern, with a larger and earlier increase in pupil dilation rate evoked by CA events.

**Figure 4.**
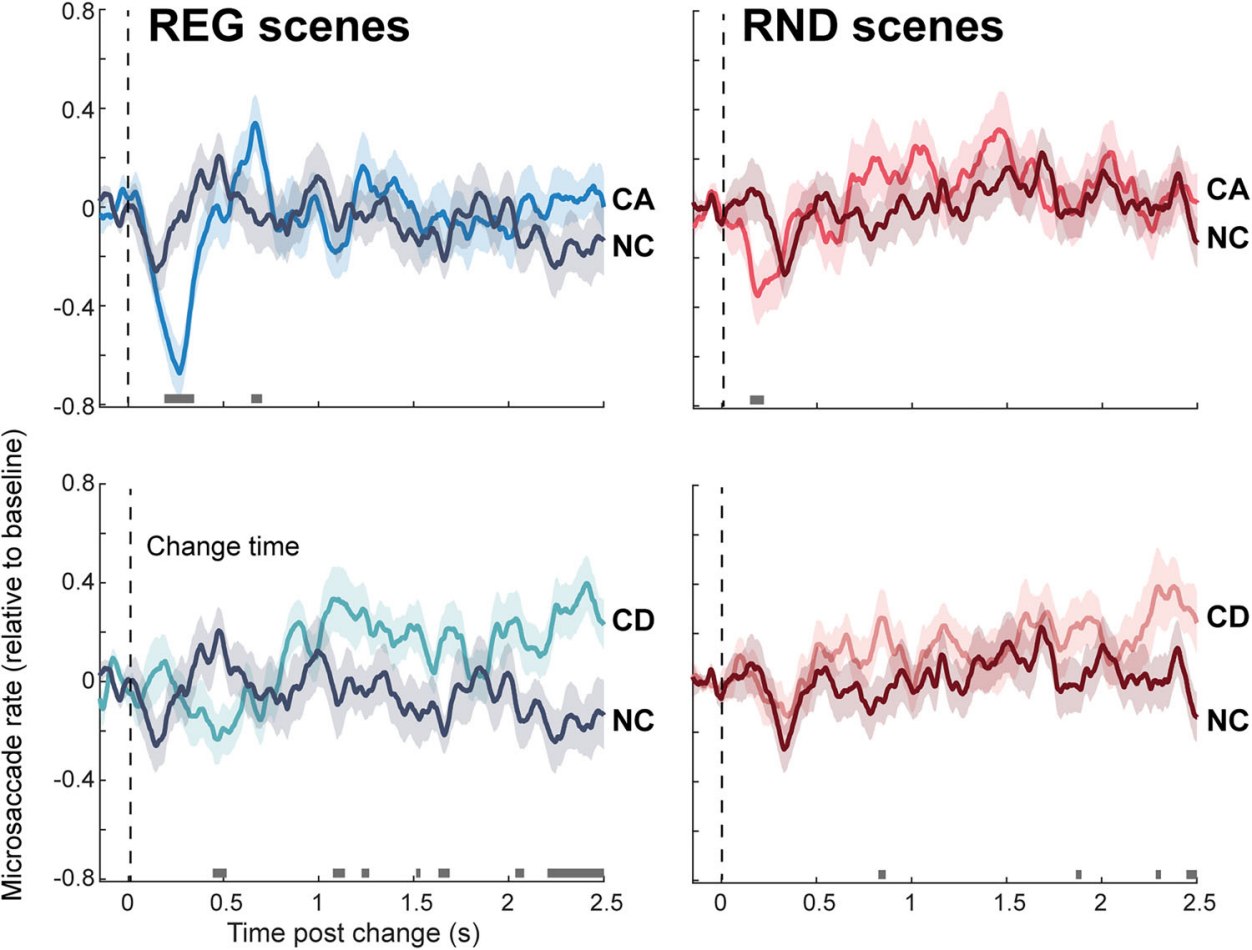
Change-evoked microsaccade responses. Baseline corrected (−0.15 to 0 s pre-onset). The shaded area shows SEM. Horizontal bars indicate significant differences (*p* < 0.05) between condition pairs. Effect sizes (Wilcoxon *r*) were calculated from the mean score at significant time points using nonparametric Wilcoxon signed-rank tests. For MS rate responses, effect sizes were *r* = 0.57 for CA versus NC in REG and *r* = 0.37 for CA versus NC in RND (only the CA conditions were examined for this, as CD-related differences were generally weaker and occurred later in time).

MS data are shown in Figure 4. A clear MSI response is visible for CA, though substantially larger in REG relative to RND scenes. For CD, a first difference between conditions emerges much later, at ∼0.5 s post-onset. This is only seen in REG scenes; no differences are observed in RND scenes.

**Figure 5.**
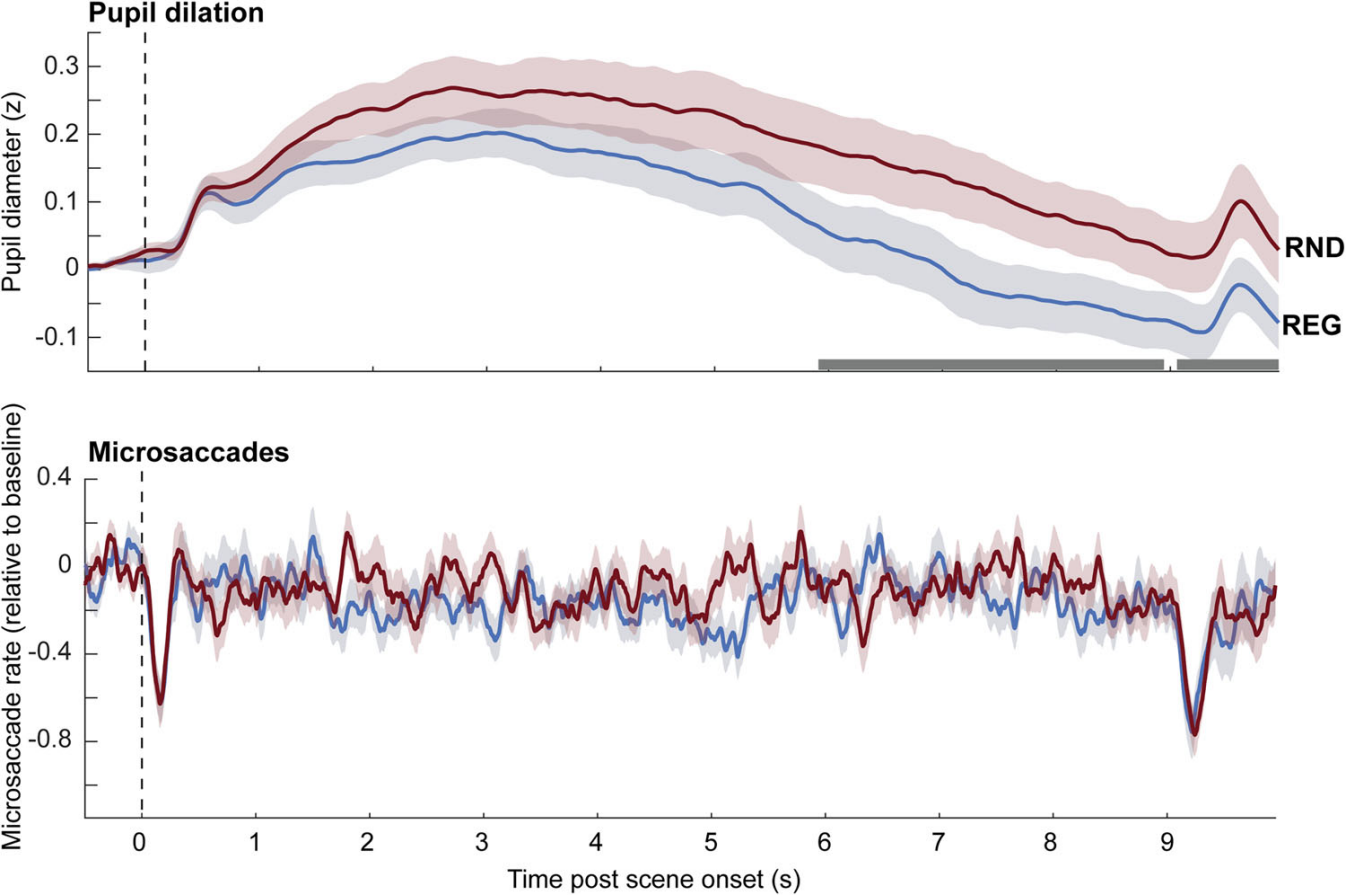
Sustained pupil dilation and microsaccades responses to regular (REG) versus random (RND) scenes. The shaded area shows SEM. The horizontal bars show time intervals during which significant differences were observed between conditions (*p* < 0.05). See Figure S2 for further control analyses confirming the robustness of these effects. Effect sizes (Wilcoxon *r*) were calculated from the mean score at significant time points within the 10 s analysis window using nonparametric Wilcoxon signed-rank tests. For PD responses, the comparison between REG and RND conditions yielded an effect size of *r* = 0.35.

A small deflection in MS rate is visible following change onset during the NC stimulus in both regularity conditions. Although this could suggest expectancy, similar deflections occurred elsewhere in the NC period (Fig. 5), implying noise may be a more likely explanation, but warranting further study.

Additionally of interest is the apparent emerging sustained difference between CD and NC whereby the MS rate in CD trials exhibits a sustained rise over the NC rate. This incidental finding is difficult to interpret given the relatively weak significance but might indicate increased scene exploration in CD scenes, as discussed further below.

### Tonic pupil dilation reveals sensitivity to scene regularity

Figure 5 shows pupil responses to REG and RND scenes (no change). Both conditions revealed a pupil diameter increase shortly after scene onset, reaching an initial peak at 0.8 s post-onset, followed by a broader peak ∼3 s after onset. Thereafter, the response entered a sustained phase, which lasted until scene offset and was associated with a slow, continuous decrease in pupil diameter. Responses to REG and RND scenes overlapped initially but diverged after 3 s post-onset, with REG scenes characterized by a faster decrease in pupil size than RND scenes. This pattern is consistent with what was previously shown by Milne et al. (2021) for regularly repeating versus random tone-pip patterns. The results thus demonstrate that listeners’ arousal level, as reflected by pupil size change, is modulated by the regularity of the complex auditory scene.

A key question concerns whether these sustained differences in pupil dilation reflect active attention directed toward the stimulus due to the gap-detection task or instead arise from more automatic, stimulus-driven processes. This question is difficult to resolve with the present data, or indeed, in general, because even during nominally passive listening, one condition may capture attention more strongly through bottom-up mechanisms. Previous MEG work (Sohoglu and Chait, 2016a) has demonstrated that sustained MEG differences between REG and RND scenes are comparable under both active and passive listening conditions. In line with this, we hypothesize that the sustained PD effects observed here reflect downstream consequences of these automatic, stimulus-driven neural processes, rather than direct modulation by task engagement. To further assess whether the sustained pupil response was influenced by performance in the decoy (gap-detection) task, we examined correlations between reaction time (RT) differences and the sustained pupil difference between REG and RND conditions. RT was used as an index of task difficulty, given its reduced susceptibility to ceiling effects. RT differences (RND − REG) from the gap-detection task were correlated with sustained pupil differences (RND − REG), both across the full analysis window (−1 to 10 s) and by taking the average between 6–10 s where we found a sustained regularity effect. No significant correlations were observed at any time point (maximum *r* = 0.28, minimum *p* = 0.1175) or with average PD difference between 6–10 s (*r* = −0.01, *p* = 0.945), indicating that the sustained pupil response was unlikely to reflect differences in gap-detection performance.

A similar analysis on MS data (Fig. 5, bottom) did not reveal any differences between conditions. Clear MSI are seen following sequence onset and offset but the two conditions do not exhibit systematic differences.

### Microsaccades, but not pupil responses, to appearing and disappearing task-irrelevant events are modulated by scene regularity

**Figure 6.**
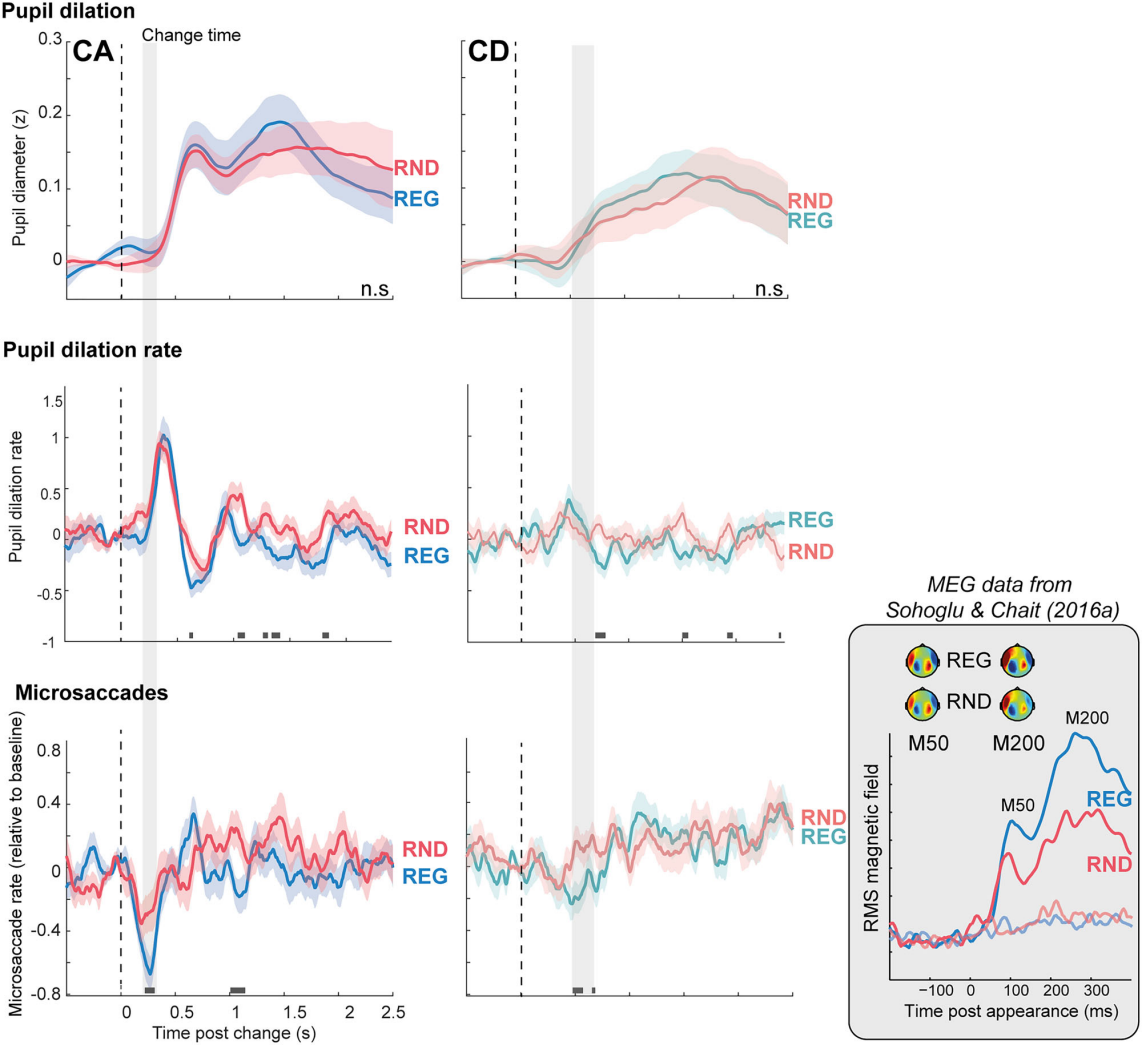
Effect of scene regularity on change-evoked microsaccade, pupil dilation, and pupil dilation rate responses. Shading around the traces indicates SEM. Horizontal bars indicate intervals where a significant difference (*p* < 0.05) is present between condition pairs. Gray shading marks the MSI effects in CA and CD scenes, highlighting the absence of corresponding differences in PD or PDR responses. Note that the PD responses represent difference waveforms (CA–NC; CD–NC; see main text). The inset shows MEG data adapted from Sohoglu and Chait (2016a), illustrating enhanced responses to CA in REG compared with RND scenes from ∼70 ms post-onset. The latency of the MSI effect for CA aligns broadly with the timing of these MEG effects. Effect sizes (Wilcoxon *r*) were calculated from the mean score at significant time points using nonparametric Wilcoxon signed-rank tests. For MS responses to CA, the comparison between REG and RND conditions yielded an effect size of *r* = 0.40.

Figure 6 plots PD, PDR, and MS responses to CA (left) and CD (right) in REG relative to RND scenes. Since REG and RND scenes are characterized by different sustained (tonic) pupil dilation baselines (Fig. 5), change-evoked responses were quantified by subtracting PD responses to the control (no change; NC) condition from the PD response to CA/CD. PD responses to both change types did not differ between REG and RND scenes.

Similarly, no effect of regularity was observed in the PDR responses.

In contrast, MS responses to CA/CD demonstrate a clear effect of regularity. CA in REG scenes are associated with a larger MSI response (more microsaccadic inhibition) than RND scenes. Significant differences between conditions emerge between 190 and 340 ms post-change onset. The MSI response appears to be triggered at the same time in both conditions but reaches a substantially lower trough in REG, indicative of a stronger attentional capture. The timing is similar to that observed in Zhao et al. (2024). An additional significant interval is observed between 1 and 1.3 s potentially reflecting that the appearing source in REG scenes—typically more perceptually salient—captured greater attention than in RND scenes.

A difference between REG and RND conditions was also observed for CD scenes, though it emerged substantially later than for CA—approximately between 0.47 and 0.68 s. This effect was accompanied by a more pronounced MSI response to CD in REG scenes. However, as noted above, the MSI response to CD is generally weaker and occurs later compared with that for CA.

## Discussion

Task-irrelevant changes in complex acoustic scenes elicit increased arousal and instantaneous attentional capture, reflected in phasic pupil dilation (PD) and MSI. Sustained PD was reduced in regular (REG) compared with random (RND) scenes, suggesting that greater predictability is linked to lower arousal. Predictability also modulated MSI, with stronger attentional capture in REG scenes, though phasic pupil responses were unaffected.

Taken together, these findings highlight a dynamic interplay between arousal, as reflected in PD, and attentional capture, as indexed by MS, during auditory scene analysis.

### Pupil response dynamics to scene changes mirror previously observed MEG responses

PD is a well-established proxy for activity in the locus ceruleus–norepinephrine (LC-NE) system, which regulates arousal and attention (Aston-Jones and Cohen, 2005; Sara and Bouret, 2012; Joshi et al., 2016; Joshi and Gold, 2020). Phasic PD responses to unexpected events are thought to reflect transient LC-NE activity (Bala and Takahashi, 2000; Wang et al., 2014). Here, we show that task-irrelevant auditory changes—source appearances (CA) and disappearances (CD)—evoke such responses. The response to CD is slower and smaller than to CA, mirroring previous MEG findings (Sohoglu and Chait, 2016b).

This timing difference may reflect the greater computational demands of detecting disappearances. While appearances can be detected from the onset of energy in a previously silent frequency band, disappearances require continuous monitoring and comparison with prior acoustic states (Yamashiro et al., 2009; Cervantes Constantino et al., 2012; Andreou et al., 2015). Alternatively, the appearance advantage may stem from ecological relevance: threats often manifest as new events, such as the sudden presence of a predator. The onset of sound thus provides rapid cues that something in the environment has changed, prompting immediate behavioral responses like freezing or orienting, whereas sound disappearance is often less urgent. These considerations suggest that the brain may rely on distinct mechanisms for processing appearances versus disappearances. Our pupil data support this view, showing that these perceptual asymmetries, also seen in brain responses, are mirrored in phasic arousal responses.

Interestingly, the temporal profile of the pupil response closely resembles that of the MEG signal. The MEG response to CA exhibits two peaks—at ∼40 and 96 ms post-onset—believed to reflect, respectively, the neural response to the transient acoustic event and subsequent processes such as recognition or attentional capture (Sohoglu and Chait, 2016b). The observation of similarly biphasic dynamics in the pupil response suggests that these distinct neural processes may have temporally dissociable effects on arousal.

### Microsaccade inhibition dynamics reveal attentional capture by scene changes

Microsaccades (MS) have gained attention in auditory research, with evidence showing that MS rates are modulated by auditory attention (Widmann et al., 2014; Abeles et al., 2020; Contadini-Wright et al., 2023). Even early microsaccadic inhibition (MSI)—a rapid reduction in MS rate following abrupt sensory events (Rolfs et al., 2008; Rolfs, 2009; Hafed et al., 2021)—is influenced by higher-order auditory factors (Kadosh and Bonneh, 2022; Zhao et al., 2024). Zhao et al. (2024) found that MSI was stronger and longer for attended versus unattended sounds, suggesting that high-level auditory processing interacts with oculomotor control.

Here, we show that task-irrelevant auditory scene changes modulate MS activity, indicating bottom-up attentional capture. Unlike the strong MSI evoked by source appearance (CA), disappearance (CD) elicits a weaker, delayed MS response—consistent with reduced behavioral sensitivity to CD (Cervantes Constantino et al., 2012; Aman et al., 2021). A gradual increase in MS rate for CD relative to NC also emerges later in the trial, particularly in REG scenes, potentially reflecting an effort to resolve complex scene changes. Behavioral evidence shows that even when listeners detect the disappearance of a source, they often fail to identify which source has disappeared (Cervantes Constantino et al., 2012). These MS dynamics may therefore reflect an automatic, information-seeking response (Schneider et al., 2020), with attentional resources shifting toward visual exploration, resulting in increased MS activity.

### Scene regularity is associated with pupil dilation-indexed reduced arousal but not with microsaccade-indexed attentional capture

Sustained pupil diameter was reduced in REG compared with RND scenes, consistent with previous findings by Milne et al. (2021) using tone sequences. This aligns with the hypothesis that predictable patterns ease processing demands, thereby lowering cognitive load and reducing arousal-as reflected in smaller pupil size. In REG scenes, listeners can likely anticipate upcoming events within each stream, facilitating more efficient processing. In contrast, the unpredictability of RND scenes places greater demands on cognitive resources.

Notably, the pupil diameter difference between REG and RND scenes emerged relatively late—approximately 6 s after sequence onset—mirroring the timing reported by Milne et al. (2021). This delayed effect contrasts with earlier differentiation seen in M/EEG data (e.g., ∼400 ms post-onset; Sohoglu and Chait, 2016a), suggesting that pupil responses reflect the downstream impact of predictability on arousal rather than the initial detection of regularity.

Conversely, sustained MS rates did not differ between REG and RND scenes. Given the established link between sustained MS activity and heightened cognitive engagement (Dalmaso et al., 2017; Lange et al., 2017; Xue et al., 2017; Abeles et al., 2020; Contadini-Wright et al., 2023), this null result suggests that scene regularity does not modulate attentional capture. This finding is particularly relevant to ongoing debates surrounding the role of predictability in guiding attention (Feldman and Friston, 2010; Zhao et al., 2013; Southwell et al., 2017; Press et al., 2020). If REG scenes are more attentionally demanding in a bottom-up manner, we would expect to see corresponding differences in MS activity, as observed in studies of stimulus-driven attention. The absence of such differences here implies that attentional engagement was comparable across both conditions.

Taken together, these findings suggest that while scene regularity reduces arousal and potentially liberates processing resources, it does not produce consistent changes in attentional allocation.

### Microsaccade inhibition, but not pupil dilation, is affected by bottom-up auditory attentional capture

Consistent with the idea that sensitivity to statistical structure supports efficient interaction with the environment (Winkler et al., 2009; Bendixen et al., 2010; Bendixen, 2014), prior studies have shown that change detection is more effective in structured (REG) than in random (RND) environments—reflected in faster reaction times and higher *d*’ values (Aman et al., 2021; de Kerangal et al., 2021).

Consistently, in Sohoglu and Chait (2016a), using stimuli essentially identical to the ones used here, change-evoked neural responses were significantly stronger in REG scenes (Fig. 6). These findings support the notion that the auditory system automatically constructs precise models of the acoustic environment based on statistical regularities. Violations of these models—i.e., unexpected events—generate prediction errors, which elicit stronger neural responses and enhance perceptual salience (see also Garrido et al., 2013; Southwell and Chait, 2018; SanMiguel et al., 2021).

We observed phasic pupil responses to changes in both REG and RND scenes. However, unlike the MEG data, the pupil measures—both pupil size (PD) and dilation event rate (PDR), the latter proposed as a closer proxy of LC firing (Joshi et al., 2016)—showed no evidence of a “regularity advantage.” While null effects should be interpreted with caution, the pattern of results suggests that any effect of regularity on PD is minimal. Notably, prior work investigating attention-related effects on PD (Zhao et al., 2024) found robust effects with a comparable sample size, indicating that regularity-related effects—if present—should have been detectable in the current dataset. This lack of modulation in the PD data implies that the “predictability advantage” observed in behavior and neuroimaging may not be mediated by changes in arousal.

In contrast, a clear effect of regularity was observed in the MS data. CA and CD events in REG scenes were associated with stronger MSI, indicating heightened attentional capture by changes in predictable contexts. Notably, the timing of this modulation—emerging subsequent to the initial sharp drop in MS incidence—closely matches the pattern reported by Zhao et al. (2024) for top-down attention. This reinforces the hypothesis that the earliest phase of MSI is not modulated by attention and that attentional effects emerge at or after the MSI trough—an observation that can help constrain models of the underlying neural circuitry.

Interestingly, the timing of the MSI effect also aligns broadly with the MEG findings, where scene predictability influenced CA-evoked responses from ∼70 ms post-change under passive listening conditions. This suggests that the MEG and MS signals may reflect related components of a shared underlying process. In the MEG data, regularity effects were localized to a network including auditory areas in the superior temporal lobe and the left postcentral gyrus. It would be important for future work to elucidate the relationship between this auditory network and the MS-linked oculomotor systems.

### Divergence between microsaccade and pupil dilation results suggests interplay between arousal and attention during auditory change detection

The divergence between MS and PD effects underscores that these measures likely capture distinct facets of cognitive processing. For change detection, it is plausible that the influence of regularity—presumably tied to rapid prediction error processing—primarily manifests as attentional capture. This form of engagement may be sufficient to support behavioral adaptation without necessitating elevated arousal levels.

Conversely, at the whole-scene level, a dissociation was observed in the opposite direction: PD, but not MS, reflected sensitivity to scene regularity (as discussed above), suggesting that a predictable context reduces arousal without inducing changes in attention. While further research is necessary to unpack the nature of this dissociation, the current findings contribute to growing evidence that PD and MS serve as complementary readouts of separate stages in scene analysis. Specifically, they appear to index dissociable effects of attention and arousal within the broader framework of automatic auditory scene analysis.

## Data Availability

The data reported in this manuscript are available at https://doi.org/10.5522/04/30531281.
